# Comparison of Clinical Outcomes Between Nonoperative Treatment and Arthroscopically Assisted Stabilization in Patients With Acute Rockwood Type 5 Acromioclavicular Dislocation

**DOI:** 10.1177/23259671241289117

**Published:** 2024-11-14

**Authors:** Doruk Akgün, Henry Gebauer, Alp Paksoy, Larissa Eckl, Agahan Hayta, Ata Ücertas, Nicolas Barthod-tonnot, Rony-Orijit Dey Hazra, Lucca Lacheta, Philipp Moroder, Jonas Pawelke

**Affiliations:** † Center for Musculoskeletal Surgery, Charité–University Medicine Berlin, Berlin, Germany; ‡ Department of Sports Orthopedics, Technical University of Munich, Munich, Germany; § Schulthess Klinik, Zurich, Switzerland; Investigation performed at Center for Musculoskeletal Surgery, Charité–University Medicine Berlin, Berlin, Germany

**Keywords:** acromioclavicular dislocation, Rockwood type 5, acute, nonoperative treatment, suture-button technique

## Abstract

**Background:**

Currently, Rockwood type 3 acromioclavicular (AC) joint dislocations are initially treated nonoperatively, whereas surgery is recommended for Rockwood type 5 dislocations. However, multiple studies have been published favoring nonoperative approaches in patients with high-grade Rockwood injuries.

**Purpose:**

To compare the clinical and radiological outcomes of patients with acute Rockwood type 5 AC joint dislocations treated nonoperatively versus with arthroscopically assisted stabilization.

**Study Design:**

Cohort study; Level of evidence, 3.

**Methods:**

Included were 48 patients with acute Rockwood type 5 dislocation who were initially treated nonoperatively between June 2010 and June 2022 and 48 patients matched according to age, sex, affected side, and follow-up interval who underwent arthroscopically assisted coracoclavicular (CC) stabilization using a suture-button technique, with additional percutaneous AC tape cerclage. Clinical outcomes were assessed based on the Subjective Shoulder Value, Nottingham Clavicle Score, Constant score, and visual analog scale for pain. The radiographic assessment included the CC distance, CC difference ratio, and degree of horizontal instability at final follow-up (62 ± 43 months).

**Results:**

At the final follow-up, the Constant score was significantly higher in the nonoperative group (*P* = .02). The operative group had significantly higher pain levels on palpation of the AC joint (1.2 ± 2.2 vs 0.19 ± 0.5 for the nonoperative group; *P* = .003). In the operative group, the mean CC difference ratio was significantly higher at the latest follow-up compared with postoperatively (1.3 ± 0.3 vs 0.67 ± 0.3, respectively; *P* < .001), whereas the CC difference ratio of the nonoperative group was significantly reduced at the latest follow-up compared with postinjury (2.0 ± 0.5 vs 2.6 ± 0.8, respectively; *P* < .001). The operative group had a significantly lower CC difference ratio compared with the nonoperative group at final follow-up (*P* < .001). More than half of the patients (56%) who were treated operatively had a loss of reduction resulting in a Rockwood type 3 state at the latest follow-up, whereas 54% of patients treated nonoperatively had spontaneous reduction of injury severity from Rockwood type 5 to Rockwood type 3.

**Conclusion:**

Although 15% of the nonoperatively treated patients eventually required surgery, successful nonoperative treatment showed similar outcomes to initial operative treatment in patients with acute Rockwood type 5 dislocation.

Dislocation of the acromioclavicular (AC) joint is a frequently observed injury, affecting primarily young males and accounting for 3% to 12% of all shoulder injuries.^11,14^ The Rockwood classification system categorizes these injuries based on the severity of dislocation, ranging from type 1 to type 6. Treatment strategies for AC joint dislocations are primarily determined by the severity of the injury (Rockwood grade). Over the years, a wide array of surgical methods have been proposed, with >150 different techniques described since the introduction of the Cadenet stabilization approach for AC joint instabilities.^6^ While there is a consensus on nonoperative management for Rockwood type 1 and 2 injuries, operative indications for type 3 and 5 injuries remain a topic of ongoing debate.^17^ Currently, the majority of the Rockwood type 3 injuries are initially treated nonoperatively, whereas surgery is recommended for Rockwood type 5 dislocations.^5,6,17,32,35^ A few studies with evidence levels 1 and 2 and multiple studies with lower levels of evidence have been published that favor nonoperative approaches in patients with high-grade Rockwood injuries (types 3 and 5).^5,8,9,20,23,25,30,35^ A recent Cochrane review comprising 6 small trials concluded, based on low-quality evidence, that surgical intervention may have no additional benefits in terms of function, return to sports, and quality of life in patients with acute high-grade Rockwood injuries^34^; however, a subgroup analysis relating to type of injury could not be conducted because of insufficient data. A recent randomized controlled trial comparing acute high-grade Rockwood type AC joint dislocations treated either nonoperatively or with a hook plate demonstrated excellent outcomes and patient satisfaction at a 24-month follow-up, regardless of the treatment modality.^8^ However, the hook plate is associated with high complication rates and significantly lower clinical scores as well as lower return-to-sports rates in patients with high-grade AC joint injuries compared with suture-button reconstruction techniques.^7,27-29,33^

There is a need for comparative studies focusing on patients with acute high-grade (Rockwood type 5) injuries, evaluating outcomes between newer suture-button fixations and nonoperative approaches. The purpose of this study was therefore to evaluate the clinical and radiological outcomes of patients with acute Rockwood type 5 AC joint dislocations treated nonoperatively and compare them with the outcomes of patients who underwent arthroscopically assisted stabilization using the suture-button technique. We hypothesized that the nonoperative group would achieve similar clinical improvements when compared with the operative group.

## Methods

### Study Design and Patient Selection

Approval from our institutional ethics committee was obtained before the onset of investigation. We conducted a retrospective review of all patients with acute Rockwood type 5 AC joint dislocation at a single center who were initially treated nonoperatively between June 2010 and June 2022. Inclusion criteria were age ≥18 years, minimum follow-up of 12 months, no previous ipsilateral injury, no concomitant injury of the ipsilateral shoulder, and available radiological data including radiographs of both shoulders to be able to assess the AC joint injury according to the Rockwood classification. Indications for nonoperative treatment were patient desire against surgery, late clinical presentation of the patient ruling out acute surgical stabilization (>3 weeks), or contraindications for operative management (high comorbidity, wounds around surgical approach, and ongoing infection). In total, 136 patients were identified in our database. Of these, 46 patients could not be contacted, 20 patients underwent secondary stabilization surgery, and 3 patients were deceased, leaving 67 patients for further analysis. Of these, 14 patients declined to participate in the study; therefore, 53 patients were ultimately included as the nonoperative group.

Patients in the nonoperative group were then matched according to age (within 5 years), sex, affected side, and follow-up time to 214 patients with acute Rockwood type 5 AC dislocation who underwent arthroscopically assisted stabilization during the study period using a suture-button system (TightRope system; Arthrex) with an additional percutaneous AC tape cerclage and who met the following inclusion criteria: (1) no concomitant injury in the ipsilateral shoulder, (2) no previous ipsilateral shoulder injury, and (3) no revision surgery after primary AC joint stabilization. Five patients from the nonoperative group could not be matched according to the criteria, leaving 96 patients (48 patients in nonoperative and 48 patients in operative group) for further analysis.

Patients who underwent nonoperative treatment were advised to use a sling for 1 to 2 weeks, allowed free range of motion as well as weightbearing as tolerated, and recommended to undergo at least 6 weeks of physical therapy to strengthen scapulothoracic musculature. Fifteen (31%) patients in the operative group who were treated before 2017 received a double-button fixation, and the remaining 33 patients who were treated after 2017 received a single-button fixation as previously described.^16,24^ All patients who underwent operation had an additional percutaneous AC cerclage with a FiberTape (Arthrex). All patients with operative stabilization were required to immobilize their operated shoulder for the first 6 weeks using an abduction pillow. Passive mobilization exercises of up to 45° of flexion and abduction were allowed in the first 3 weeks and up to 90° in the following 3 weeks. Free passive and active range of motion exercises were started at week 7 postoperatively.

### Radiological and Clinical Evaluations

The clinical outcome was assessed based on the functional measures of the Subjective Shoulder Value (SSV)^19^; Nottingham Clavicle Score (NCS)^10^; Constant score (CS)^13^; and visual analog scale for pain at rest, during motion, and with palpation. Furthermore, the sport activity level of the patients was evaluated preoperatively as well as at the latest follow-up, and the time to return to sports was reported. Patients were also asked if they noticed a cosmetic difference between both AC joints and, if so, whether it disturbed them.

Postinjury panoramic radiographs with a 10-kg load were obtained in all patients. For patients who underwent surgery, panoramic radiographs without load were routinely obtained on postoperative day 2 to evaluate initial reduction of the AC joint. The final radiographic assessment for both study groups included panoramic radiographs with a 10-kg load and Alexander views^1^ of both shoulders to measure the coracoclavicular (CC) distance and evaluate the degree of horizontal instability, respectively. Rockwood type 3 injury is defined as an increase in the CC distance by 25% to 100% compared with the uninjured side, and type 5 injury as an increase by >100%.^31^ The CC difference ratio was calculated as CC distance of the injured side to CC distance of the uninjured side. The AC joint was classified as stable if the clavicle was in line with the acromion on the Alexander view. Partial dynamic posterior translation (DPT) was defined as incomplete posterosuperior displacement of the clavicle in relation to the acromion, and complete DPT was defined as no contact between the joint surfaces.^26^

### Statistical Analysis

The Kolmogorov-Smirnov test was used to test for normal distribution. The 2-sample *t* test (for parametric distributions) or Mann-Whitney *U* test (for nonparametric distributions) was used to compare continuous variables between the operative and nonoperative groups. The results are reported as means with standard deviations or as frequencies with percentages. Furthermore, associations between radiographic parameters (CC difference ratio and DPT) and clinical results (SSV, NCS, and CS) were performed using the Pearson correlation. For statistical analyses, IBM SPSS Statistics software (Version 29.0; IBM) was used. *P* <.05 was considered statistically significant.

## Results

### Study Population

The mean final follow-up for the study cohort was 62 ± 43 months. There were no significant differences between the nonoperative and operative groups in terms of age, sex, affected side, or follow-up time (Table 1).

**Table 1:** Demographic Comparison of the Study Cohort^*a*^

Variable	Nonoperative (n = 48)	Operative (n = 48)	*P*
Age, y	41.2 ± 14	42.4 ± 14	.12
Sex, female/male	1/47	1/47	≥.99
Affected side, left/right	19/29	19/29	≥.99
Follow-up, mo	61 ± 43	63.1 ± 43	.10

a Data are presented as mean ± SD or No. of patients.

### Clinical Findings

At the final follow-up, there was a significantly higher CS in the nonoperative group; however, it did not reach the minimal clinically importance difference of 10.4 points^21^ (Table 2). Patients in the operative group reported significantly greater pain during palpation of the AC joint versus the nonoperative group (1.2 ± 2.2 vs 0.19 ± 0.5; *P* = .003) as well as greater but not significantly higher pain during movement (1.2 ± 2 vs 0.6 ± 1; *P* = .08). In the operative group, 88% (42/48) of patients reported returning to their preinjury level of sports, including overhead sports in 71% (30/42), which was similar to rates in the nonoperative group (85% [41/48] and 71% [29/41], respectively). Although the return-to-sports rate did not differ between groups, patients treated nonoperatively returned to sports significantly faster than patients treated operatively (2.8 ± 2.2 vs 6.4 ± 3.3 months, respectively; *P* < .001) (Table 2).

**Table 2:** Comparison of Outcomes Between the Nonoperative and Operative Groups^*a*^

Outcome	Nonoperative (n = 48)	Operative (n = 48)	*P*
Subjective Shoulder Value	90.6 ± 11	87.5 ± 16	.20
Nottingham Clavicle Score	91.3 ± 9	88.2 ± 14	.20
Constant score	92.3 ± 10	87.7 ± 12	**.02**
VAS pain score			
At rest	0.17 ± 0.8	0.42 ± 1.2	.20
During movement	0.6 ± 1	1.2 ± 2	.08
With palpation	0.19 ± 0.5	1.2 ± 2.2	**.003**
Return to sports, mo	2.8 ± 2.2	6.4 ± 3.3	**<.001**
Return-to-sports rate	41/48 (85)	42/48 (88)	.10

a Data are reported as mean ± SD or n/total available (%). Boldface *P* values indicate a statistically significant difference between groups (*P* < .05). VAS, visual analog scale.

Most of the patients were satisfied with the cosmetic appearance of their injured shoulder at final follow-up. In the operative group, 56% of the patients reported cosmetic concerns but only 15% of these patients were dissatisfied with the cosmetic differences. In the nonoperative group, 75% of the patients reported cosmetic concerns but only 8% of these patients were dissatisfied.

### Radiological Findings

The CC difference ratio at the time of injury was 2.6 ± 0.8 in the nonoperative group and 2.6 ± 0.6 in the operative group (*P* = .9), respectively. Of the 48 patients who underwent surgery, 13 (27%) had an anatomically reduced AC joint, 32 (67%) had an overreduced AC joint, and 3 (6%) an incompletely reduced AC joint 2 days postoperatively, with a mean CC difference ratio of 0.67 ± 0.3. The mean time from injury to surgical stabilization was 8.3 ± 4 days. Eleven patients in the nonoperative group and 4 patients in the operative group refused to undergo radiographic follow-up evaluation and were excluded from further radiological analysis. The mean CC difference ratio of the operative group was significantly higher at the latest follow-up compared with the immediate postoperative CC difference ratio (1.3 ± 0.3 vs 0.67 ± 0.3, respectively; *P* < .001), whereas the CC difference ratio of the nonoperative group was significantly reduced at the latest follow-up compared with postinjury (2.0 ± 0.5 vs 2.6 ± 0.8; *P* < .001). The operative group had a significantly lower CC difference ratio compared with the nonoperative group at the final follow-up (*P* < .001).

**Figure 1.**
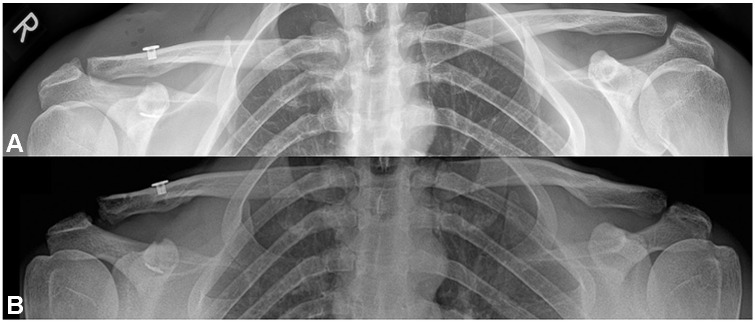
(A) Panoramic radiograph without load on postoperative day 2 after single TightRope stabilization showing an overreduction of the right acromioclavicular joint. (B) Panoramic radiograph with 10-kg load at final follow-up showing a loss of reduction of the right acromioclavicular joint compared with the postoperative radiograph.

After excluding 3 patients with an incomplete postoperative reduction, 56% (23/41 patients) of the operatively treated AC joints that were initially anatomically reduced or overreduced turned into Rockwood type 3 state at the latest follow-up (Figure 1). A horizontally stable joint was evident in 32%, whereas partial DPT was observed in 57% and complete DPT in 11% of all patients in the operative group at the final follow-up.

**Figure 2.**
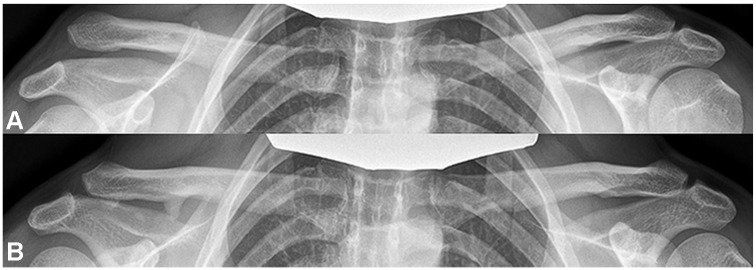
(A) Panoramic radiograph with 10-kg load at the time of injury showing a Rockwood type 5 dislocation on the right acromioclavicular joint. (B) Panoramic radiograph with 10-kg load at final follow-up showing a spontaneous reduction of the injured acromioclavicular joint after nonoperative treatment.

In patients treated nonoperatively, 54% (20/37) had spontaneous reduction of the injury severity from Rockwood type 5 to Rockwood type 3 (Figure 2). A horizontally stable joint was evident in 5%, whereas partial DPT was observed in 41% and complete DPT in 54% of the patients in the operative group at the final follow-up.

### Association of Clinical and Radiological Findings

There were no significant associations between the CC difference ratio at the latest follow-up and the SSV (*P* = .9), NCS (*P* = .93), or CS (*P* = .73). DPT also did not show any correlations with SSV (*P* = .3), NCS (*P* = .92), or CS (*P* = .2).

## Discussion

The most important finding of this study was that similar clinical results were able to be achieved with both nonoperative and operative treatment in patients with acute Rockwood type 5 AC joint dislocation. Our study results are in line with the recent literature. Two recent randomized controlled trials including patients with acute types 3 and 5 AC joint dislocations, comparing hook plate with nonoperative treatment, reported that shoulder function was well restored and patients were satisfied with the result at the latest follow-up regardless of treatment modality.^8,9^ Although the study by the Canadian Orthopaedic Trauma Society^9^ did not differentiate between Rockwood types when analyzing outcomes, Bostrom Windhamre et al^8^ showed similar results for patients with a Rockwood type 3 and 5 injury, regardless of treatment modality. While the hook plate is a well-known and commonly used treatment method for acute AC joint instability,^2,3^ it seems to have more complication rates, significantly lower clinical scores, a higher persistent horizontal instability rate, and a significantly lower return-to-sports rate in patients with acute high-grade AC joint injuries compared with newer suture-button reconstruction techniques.^7,27-29,33^ The present study also found no differences in clinical outcome between the suture-button reconstruction technique and nonoperative treatment in patients with Rockwood type 5 AC joint dislocations. However, it should be noted that in 15% of our nonoperatively treated patients, a secondary stabilization surgery was necessary because of failed initial nonoperative treatment. Thus, routine surgery in all patients with an acute Rockwood type 5 injury cannot be supported and would lead to overtreatment in a great subset of patients who would benefit from nonoperative treatment. There is an emerging need to find which patients experience failure with nonoperative treatment so as to treat them surgically in the acute setting.

Although the primary purpose of surgical stabilization is the anatomic restoration of the AC joint, loss of reduction seems to be very common. In our cohort, 56% of the operatively treated patients had a loss of reduction ending up in a Rockwood type 3 injury, even though 67% had an operative overreduction, as suggested by Maziak et al^26^ to achieve favorable radiological results. These authors reported loss of initial reduction in almost 75% of their patients, with only 62% having an anatomic or almost anatomic reduction of the operatively treated AC joint at the time of last radiological follow-up. This is in line with most of the current literature.^15,22^ Clavert et al^12^ performed a prospective multicenter study to assess types of failure after primary arthroscopic cortical button fixation and reported radiographic failure (defined as 50% loss of reduction) in 41% of the patients. This may be because of the reduced tissue quality of the healed ligaments and/or failure of the tightrope construct over time. In contrast to loss of reduction of the operatively treated patients, we observed a spontaneous reduction of the CC distance ratio in nonoperative treated patients. Overall, 54% of the patients with a Rockwood type 5 injury who were treated nonoperatively had a Rockwood type 3 injury at the latest follow-up. To the best of our knowledge, this is the first time this radiological finding has been described. One may speculate that this could be the result of a better scapulothoracic orientation and recovery of deltotrapezial muscle tone or soft tissue healing with scar tissue formation in the healed stage, or worsening of the CC distance in an acute setting in the case of using 10-kg loading radiographs compared with the healed stage, where the scar tissue does not allow the CC distance to increase.

Although anatomic reduction outcome was reported to correlate with the functional outcome (unweighted CS) after arthroscopically assisted stabilization of acute AC joint dislocation by Barth et al,^4^ we could not find an association between radiological parameters (CC difference ratio and DPT) and clinical scores (SSV, NCS, and CS) in the present study. This is in line with recent findings in the published literature.^26^

Patients treated nonoperatively returned to sports significantly faster than patients who underwent operative treatment in our study cohort. Similar to our results, Beitzel et al^5^ reported in their systematic review of 14 studies (706 patients) evaluating AC joint injury that patients managed nonoperatively had a quicker recovery, allowing them to return to sports faster than those treated operatively. Gawel et al^18^ analyzed the return-to-sports rate after operative management of AC joint dislocation in their systemic review. Return-to-sports timelines ranged between 2 and 12 months, the most common timeline being 6 months (40%) after surgery, which was similar to our results.

### Limitations

This study has several limitations that should be considered in interpreting the data. Most notably, there could be a selection bias because of the retrospective design. Furthermore, it was not a randomized trial, and patients desired to undergo nonoperative treatment, which may have led to their minimizing their residual symptoms. To reduce this selection bias, a matched-pair study design was chosen. Furthermore, a subset of patients could not be contacted, and 11 patients in the nonoperative group and 4 in the operative group refused to undergo radiological evaluation at the latest follow-up, which may have altered the presented results. Furthermore, our results may not be applicable for a certain subgroup of patients with specific demands, such as participation in overhead sports, who may benefit from surgery.

## Conclusion

Despite 15% of the nonoperatively treated patients eventually requiring surgery, successful nonoperative treatment showed similar outcomes to initial operative treatment in patients with acute Rockwood type 5 AC joint dislocations. Interestingly, nonoperative treatment showed an improvement in CC distance at follow-up, reducing the level of instability to Rockwood type 3 in half of the cases, whereas 50% of the patients in the operative group had a loss of reduction.
